# Evaluating undergraduate academic prerequisites across institution types in an integrated med-ical curriculum

**DOI:** 10.5116/ijme.6ab2.3ff3

**Published:** 2026-10-08

**Authors:** Christian C. Steciuch, Sarah A. Keim, David W. Becker, Brian D. Steele

**Affiliations:** 1 Department of Psychiatry and Behavioral Sciences, University of Kansas School of Medicine, USA; 2 Department of Surgery, University of Kansas School of Medicine, USA; 3 Department of Internal Medicine, University of Kansas School of Medicine, USA; 4 Department of Family Medicine & Community Health, University of Kansas School of Medicine, USA

**Keywords:** Medical school admissions, institution type, holistic review

## Abstract

**Objectives:**

This evaluation study examines how undergraduate courses, institution type, and the presence of a gap year relate to outcomes at an MD-granting medical school in the United States.

**Methods:**

Archival educational data was analyzed from 1,476 matriculating students between 2017 and 2023. Variables included undergraduate science grade point average, medical college aptitude test subscale scores, course credit hours, institution type, and gap time since graduation. Correlational analyses and mean comparison between groups were conducted.

**Results:**

Students’ grade point average in required prerequisites at four-year universities was moderately correlated with academic performance (Pearson’s r’s = .17-.29), but grade point average in the same courses at community colleges was not. The one exception was physics, (Pearson’s r = .20). Notably, the number of credit hours in required prerequisites was not correlated with medical school performance in any institution type. There were no significant differences between students who graduated from a public or private, or domestic or international university. Students who had at least one gap year (M = 83.8%, SD = 5.56%) performed slightly lower on internal block exams than students who had no gap year (M = 84.9%, SD = 5.16%), t_(1235)_ = 3.686, p < .001, Cohen’s d = .21.

**Conclusions:**

Although the findings are from a single institution, institution-level analyses are essential for dispelling commonly held beliefs about applicant readiness, giving credence to a more holistic view of applicants.

## Introduction

Medical school admission committees are charged with the annual task of evaluating thousands of applicants for a significantly smaller matriculating cohort. This process involves evaluating each student’s premedical coursework, aptitude tests like the Medical College Aptitude Test (MCAT), situational judgment tests, and how well the applicant interviews (if selected). Although some measures like the MCAT afford a more objective, comparable metric to compare students, other measures like a students’ premedical coursework can greatly vary. Even with providing a list of required prerequisite coursework, undergraduate universities can vary greatly in course content and assessment, even if the goal of the course is to prepare an undergraduate student for a medical school curriculum.^1^ The actual differences in course content and assessment are further complicated by the admissions committee’s potential assumptions of prestige of the universities offering the courses. This belief assumes that students completing courses are more selective universities have retained more prerequisite knowledge,^1^ and that smaller universities and community colleges are perceived to offer easier courses.^2^ To assess the validity of this belief, we assessed the extent that prerequisite and non-prerequisite courses across four-year universities and smaller colleges related to medical student performance.

The admissions committee at the University of Kansas School of Medicine (KUSOM) has evaluated student applicants using a holistic view of applicants by including mission-aligned attributes in addition to the historical importance of previous academic metrics. This change to a holistic application review occurred in 2017 when Active-learning, Competency-based, Excellence-driven (ACE) curriculum changed from a discipline-based block curriculum to an integrated curriculum.^3^ In this context, an integrated curriculum refers to the integration of basic and clinical science when biomedical content is delivered to students. This affords students the opportunity to integrate basic science content within a clinical context. In the ACE curriculum, learning activities were redesigned to include a large percentage of active and collaborative learning in problem-based and case-based collaborative learning sessions throughout the pre-clerkship curriculum.^3^

For more than a decade, the majority of US medical schools engage in a holistic review of applicants.^4^ Studies centered around admissions processes and procedures have shown that academic records, aptitude tests, situational judgement tests, and selection centers are effective selection methods.^5^ However, curricular structures, premedical coursework, and additional prerequisite requirements also vary across medical schools.^6^ Other non-academic aptitude criteria used for admissions include insight into medicine, extracurricular activities and interests, personality, motivation, and linguistic and communication skills.^7^ Although several studies have examined the admissions prerequisites, criteria, and processes, none have specifically examined those factors and their relationships with academic performance in an integrated curriculum.

In the context of an integrated curriculum and the holistic approach to the review of applicants, the admission requirements at KUSOM were thoroughly reviewed to determine if the current prerequisites are meeting the needs of the school and the applicants to be successful within the new curriculum. This study sought to determine if a medical student’s undergraduate course work across university type and experiences related to academic performance within the integrated curriculum. We reasoned that these admissions variables may have varying degrees of relation to academic performance in an active-learning and integrated curriculum due to the increased collaborative and integrative nature of the content delivered. To evaluate these putative relationships, we had four Aims. Aim 1 addressed the extent that a medical students’ undergraduate course credits and GPA across institution type predict success in the Phase I and Phase II ACE curriculum. Aim 2 addressed the extent that medical students’ academic aptitude at matriculation predict success in the pre-clerkship and clerkship phase of the ACE curriculum. Aim 3 addressed the extent that medical students’ undergraduate institution predicted academic performance in the pre-clerkship and clerkship phase of the ACE curriculum. Aim 4 addressed the extent that the amount of time elapsed since undergraduate graduation and starting medical school predicted student performance in the pre-clerkship and clerkship phase of the ACE curriculum.

## Methods

### Study Design and Participants

The current study examined archival data from the matriculating students from the 2017 – 2023 academic years at KUSOM. Student data was obtained from the American Medical College Application Service (AMCAS), paired with their medical education records at KUSOM, and de-identified. The study was approved as a quality improvement by the KUSOM institutional review board and approved by an internal data acquisition committee. The University of Kansas School of Medicine admits approximately 211 medical students in each cohort, spread across three campuses in Kansas. Overall, there were 1,476 students included in the final dataset. Student success outcomes included their performance on internal block exams, number of internal block exam failures, and an internal clinical skills assessment (CSA) given at the end of the clerkship year.

### Measures

Suggested Course Prerequisites Credit Hours and GPA. The suggested course prerequisites at KUSOM were: anatomy, neuroanatomy, embryology, immunology, genetics, biochemistry, and physiology. To assess Aim 1, the number of credit hours in the suggested prerequisites were summed for each student. Given the low number of suggested prerequisites for some students (0-3 credit hours; equivalent to one course), GPA was not calculated for suggested prerequisites.

Required Course Prerequisites Credit Hours and GPA. Course GPA by required content areas across institution type (four-year university or community college) was also considered. Courses from biology, chemistry, inorganic chemistry, & physics departments were chosen based on our required prerequisites. Although courses varied within each content area, we grouped courses within each area to get an estimate of each students’ preparedness for that content. For the 1,476 students in the sample, 66,630 courses were listed across these content areas. Roughly 6% of courses contained non-letter grades of ‘P’, ‘S’, ‘N’, or ‘L’. Each institution had different definitions for non-letter grades, and converting or weighting the scores could have introduced more bias to the dataset. For those reasons, the 6% of non-letter grades were excluded from the dataset. For the remaining 62,632 courses, an A was converted to 4.0, B to 3.0, C to 2.0, D to 1.0, F to 0.0. Students’ average GPA in each content area by institution type (four-year institution or junior college and community college) was calculated. We categorized course GPA by institution type to address the belief that four-year university credits are more rigorous or more difficult than community college credits.

Initial Block Exams and Initial Block Exam Failure. In the ACE curriculum students complete a block exam covering the previous two to three weeks of content every two to three weeks. The block exam questions are written in the United States Medical Licensing Examination (USMLE) format, requiring students to apply knowledge and select the ‘most correct’ answer. Both internal block exams and the clinical skills assessment below have previously demonstrated high validity with clinical knowledge and clinical skills performance.^8^ Across the first two years of the curriculum, students completed 29-31 block exams (depending on their academic year). In the event of a failure, students had two additional opportunities to pass a remediation exam. For the number of exam failures, only the initial exam failure for each block exam was considered. Across the seven cohorts included in the study, there were very few second and third remediation attempts. For that reason, the number of second and third remediation attempts were not considered.

Clinical Skills Assessment (CSA). The CSA is a 10-station clinical encounter in which students must engage in ten standardized patient encounters, each with varying presenting problems. Students take a patient history, document symptoms, perform physical exams, develop a differential diagnosis, and provide a future treatment plan. They are then assessed on a pre-determined checklist of correct clinical skills and behaviors. The CSA is administered at the end of the clerkship year. Their final score is reported as a percentage of correct items across roughly 40 items per encounter related to history taking, communication skills, documentation, and clinical reasoning. The CSA serves as a competency checkpoint for students at the end of their clerkship year, and they are required to pass to advance to their final year.

## Results

The results of each statistical test are presented below by Aim. For student success outcomes in the ACE curriculum, the overall block exam mean for all ACE cohorts was 84.35% (SD = 5.4%), the average number of initial exam failures was 1.30 (SD = 2.54), and the average CSA percentage was 69.4% (SD = 5.51%).

To assess Aim 1, the number of credit hours in suggested prerequisites were summed for each student. Each course type was then correlated with mean block exam performance, number of internal exam failures, and CSA performance. The number of course credit hours were not found to be significantly correlated with any student success outcome, p’s > .05. Most students had very few, if any, credit hours in these suggest prerequisites, potentially contributing towards these null findings. This is explored more in the Discussion.

Course GPA by required content areas across institution type (four-year university or community college) was also considered. When course GPA was compared across four-year institutions to community colleges, biology (Pearson’s r = .29), chemistry (Pearson’s r =.17), and organic chemistry GPA (Pearson’s r = .27) at four-year institutions were correlated with mean block exam performance, while biology, chemistry, and organic chemistry GPA from community colleges was not correlated with mean block exam performance. Grade point average in physics was similarly predictive of mean block exam performance across four-year institutions and community colleges (Pearson’s r’s = .18 and .20, respectively). Table 1 displays the correlations between student success outcomes across four-year institutions and Table 2 displays the correlations between student success outcomes and community college courses.

To assess Aim 2, students’ most recent total MCAT scores and undergraduate science GPA were correlated with student success outcomes. Students’ most recent total MCAT score was moderately correlated with initial block exam mean (Pearson’s r = .32, p < .01), number of initial exam failures (Pearson’s r = -.23, p < .01), and CSA score (Pearson’s r = .08, p < .01). Undergraduate science GPA was moderately correlated with initial block exam mean (Pearson’s r = .37, p < .01), number of initial exam failures (Pearson’s r = -.32, p < .01), and CSA score (Pearson’s r = .19, p < .01). Table 3 below displays the correlation matrix of MCAT subscales, and undergraduate GPA with student success outcomes.

To assess Aim 3, students’ undergraduate institution was categorized into either public or private, domestic or foreign, and four-year institution, junior college, or community college. For the private v. public and domestic v. foreign comparisons, groups were compared across each student success outcome using a Mann-Whitney’s U test, given the sample size disparities. An independent samples t-test revealed that there were no significant differences between the private and public college attending students, p > .05. Similarly, an independent samples t-test revealed that there were also no significant differences between the domestic and foreign institution attending students, p > .05.

For the four-year institution v. junior college v. community college groups, a Kruskal-Wallis test was used to compare each group across the student success outcomes given the sample size disparities. In cases where students attended multiple institution types (four-year university, college, or community college), they were grouped into the ‘higher’ category, i.e. four-year university instead of community college. The Kruskal-Wallis test determined that there were no significant differences in block exam scores, number of exam failures, or CSA score, p’s > .05. Table 4 below mean differences between college types and student success outcomes.

To assess Aim 4, students who had at least one ‘gap’ year between undergraduate graduation and matriculating into medical school were compared. These two groups were compared against one another with independent samples t-tests, with an alpha value of .05. Students who matriculated within a year of undergraduate graduation (M = 84.9%, SD = 5.16%) scored 1.1% higher on initial block exams than students with at least a year in between graduation and matriculation (M = 83.8%, SD = 5.56%), t_(1235)_ = 3.686, p < .001, Cohen’s d = .21. Students who matriculated within a year of undergraduate graduation (M = 1.10, SD = 2.20) failed 0.41 fewer initial block exams than students with at least a year in between graduation and matriculation (M = 1.51, SD = 2.84), t_(1235) _= 2.870, p = .002, Cohen’s d = .16. And independent samples t-test also revealed that there were no significant differences in CSA scores, p > .05. Table 5 displays the mean differences amongst students with those who matriculated within a year of graduation and those who matriculated more than a year post-graduation and student success outcomes.

**Table 1:** Correlation Matrix of University Course GPAs, and ACE Outcomes

Variable	1.	2.	3.	4.	5.	6.	7.
1. Biology GPA	--						
2. Chemistry GPA	.466** (1336)	--					
3. Org. Chemistry GPA	.482** (1363)	.416** (1319)	--				
4. Physics GPA	.373** (1352)	.460** (1307)	.451** (1336)	--			
5. Block Exam Mean	.291** (1186)	.168** (1132)	.271** (1160)	.183** (1149)	--		
6. Number of Exam Failures	-.226** (1186)	-.132** (1132)	-.204** (1160)	-.152** (1149)	-.777** (1237)	--	
7. CSA Score	.096** (860)	.105** (823)	.113** (840)	.084* (834)	.451** (891)	-.273** (891)	--

Note: * indicates p < .05, ** indicates p < .001. N presented in parentheses.

**Table 2:** Correlation Matrix of Junior/Community College Course GPAs, and ACE Outcomes

Variable	1.	2.	3.	4.	5.	6.	7.
1. Biology GPA	--						
2. Chemistry GPA	.035 (207)	--					
3. Org. Chemistry GPA	.430** (79)	.057 (89)	--				
4. Physics GPA	.504** (95)	-.044 (160)	.442** (80)	--			
5. Block Exam Mean	.101 (178)	.016 (1137)	.074 (79)	.208* (139)	--		

**Table 3:** Correlation Matrix of MCAT subscales, Undergraduate GPA, and ACE Outcomes

Variable	1.	2.	3.	4.	5.	6.	7.	8.	9.	10.
1. MCAT CPBS	**--**									
2. MCAT CARS	.208** (1447)	--								
3. MCAT BBFL	.514** (1447)	.263** (1447)	--							
4. MCAT PSBB	.321** (1447)	.317** (1447)	.336** (1447)	--						
5. MCAT Total	.714** (1447)	.666** (1447)	.738** (1447)	.694** (1447)	--					
6. uScience GPA	.249** (1447)	.018 (1447)	.187** (1447)	.092* (1447)	.189** (1447)	--				
7. uGPA	.19** (1445)	.007 (1445)	.14** (1445)	.089* (1445)	.146** (1447)	.936** (1471)	--			
8. Block Exam Mean	.293** (1210)	.155** (1210)	.313** (1210)	.149** (1210)	.322** (1210)	.365** (1237)	.342** (1237)	--		
9. Number of Exam Failures	-.227** (1210)	-.091* (1210)	-.223** (1210)	-.104** (1210)	-.227** (1210)	-.316** (1210)	-.298** (1237)	-.778** (1237)	--	
10. CSA Score	.058 (875)	.065* (875)	.103** (875)	.003 (875)	.083* (875)	.185** (875)	.194** (897)	.451** (891)	-.273** (891)	--

Note: * indicates p < .05, ** indicates p < .001. N presented in parentheses.

**Table 4:** Comparison of Institution type across Student Success Outcomes

Institution Type	Block Exam Mean	Number of Exam Fails	CSA Score
Public	83.9% (5.0%) (N = 719)	1.16 (1.84) (N = 719)	69.3% (5.6%) (N = 528)
Private	84.8% (4.7%) (N = 115)	1.39 (1.82) (N = 115)	69.1% (5.6%) (N = 86)
Domestic	84.1% (4.8%) (N = 812)	1.34 (1.84) (N = 812)	69.2% (5.6%) (N = 597)
International	83.5% (3.7%) (N = 22)	2.00 (0.86) (N = 22)	69.3% (5.4%) (N = 17)
Four-Year University	85.4% (4.8%) (N = 605)	1.22 (1.69) (N = 605)	69.4% (5.4%) (N = 605)
Junior College	84.2% (5.1%) (N = 50)	1.57 (2.06) (N = 50)	68.9% (6.7%) (N = 50)
Community College	85.1% (4.7%) (N = 236)	1.46 (1.70) (N = 236)	69.4% (5.4%) (N = 236)

Note: SD presented in parentheses. N presented below.

**Table 5:** Comparison of Gap Year Categories across Student Success Outcomes

Number of Gap Years	Block Exam Mean	Number of Exam Fails	CSA Score
0-1 Years	**84.9%** (5.2%) (N = 631)	**1.10** (2.19) (N = 631)	69.6% (5.2%) (N = 485)
1+ Years	**83.8%** (5.6%) (N = 606)	**1.51** (2.84) (N = 606)	69.2% (5.8%) (N = 412)

Note: SD presented in parentheses. N presented below. Bold indicates a significant mean difference.

## Discussion

The results give valuable insight into how pre-medical academic variables relate to student performance in our integrated medical curriculum. The study supports the importance of individual medical schools to evaluate the academic requirements for entering students and to consider how these requirements align with curricular success. As institutions seek innovative ways to educate future physicians, they need to evaluate which incoming requirements best support student’s success in the curriculum. The study provides a foundation for future institutional research on prerequisite factors related to integrated curricular success. It can be replicated across various institutions and tailored to individual school’s admissions needs.

One notable finding is that the number of credit hours in prerequisite science courses did not correlate with student success. Credit hours alone may be an insensitive measure of academic aptitude or preparedness. Instead, the quality and rigor of coursework appear to matter more than quantity. Student’s GPA in biology, chemistry, and organic chemistry courses taken at four-year institutions was predictive of success, while GPA in the same subjects from community colleges was not. This may reflect the inclusion of upper-level coursework at four-year institutions, which aligns more closely with the complexity and depth of medical school content. This is a critical consideration when evaluating prerequisite coursework: where science courses are taken may be as important as what courses are taken. The lack of differences in performance based on baccalaureate-granting institution type may also reflect a broader trend observed in Aim 1, student GPA is likely a more sensitive indicator of knowledge acquisition than institutional prestige. This finding supports previous literature that undergraduate institutional selectivity and research classification were not significant predictors for student performance medical school.^9^

The results suggest that specific undergraduate courses—such as anatomy, microbiology, or immunology—were not predictive of medical school performance. On the face of it, these findings challenge traditional assumptions about prerequisite coursework^10^^-^^14^ and suggest that admissions committees should focus more on demonstrated academic performance than on a list of specific courses. However, most students had very few credit hours in these courses, which likely reduced the likelihood of finding a significant relationship between the prerequisite courses and student outcomes. Previous studies have found that prerequisites positively correlate with medical school performance, but it is possible that the ACE curriculum is tailored to bring students up to speed in these content areas given that most students do not have prior experience with these courses. The results have implications for premedical advising, as students often select courses they believe will prepare them for medical school. A transparent, evidence-based assessment of institutional requirements can help students make informed decisions. The variety of premedical course requirements across medical schools may create confusion,6 but institutional self-assessment ensures that each school can clearly articulate what is needed for success in its curriculum.

National data and research on premedical preparation for medical school are valuable, but this study highlights the importance of institutional-level analysis specific to the curriculum. Each medical school must evaluate their own admissions criteria and student outcomes to ensure alignment. There can still be translational errors from applying the results of a multi-institutional study to one’s singular institution if the curricular structures significantly differ. The results of this study should inform not only admissions decisions but also curriculum design, student support services, and faculty development. Institutional research of this kind can provide actionable feedback to multiple stakeholders including Admissions Committees, Curriculum Committees, Student Affairs, and Offices of Educational Affairs, who often operate in silos despite their shared goal of supporting student success.

Consistent with literature about MCAT and GPA data,^15^^, ^^16^ Aim 2 found that students’ most recent total MCAT scores and undergraduate science GPA had moderate relationships with academic performance. The BBFL and CPBS MCAT sections demonstrated stronger relationships with academic outcomes than the CARS and PSBB sections. This is relevant for admissions committees that rely heavily on the total MCAT score. A holistic interpretation of MCAT subsection performance could help identify students with strong potential, even if their total score is lower.

Aim 4 revealed that students who matriculated within one year of graduation performed slightly better in the curriculum, suggesting that academic momentum may influence early medical school performance. The finding could support that time away from academics can lead to greater information loss, making it harder to recall foundational knowledge in medical school. Medical students who have taken time off before starting medical school could also have additional factors more common among older students influencing their success, including increased family obligations, childcare responsibilities, and reduced parental support. As more students take gap or growth years before entering medical school,^17^ institutions must consider how to help incoming students regain academic momentum. These findings complement previous research on the social benefits of gap years^18^ and align with recent literature on the educational challenges and opportunities associated with time away from school.^17^^, ^^19^ Further research should explore how time away from academics affects performance in integrated curricula and how schools can support students during this transition.

### Limitations

Although this study is limited by its single-institution scope, the findings remain meaningful and actionable. The analysis did not account for additional factors such as student support systems or the broader range of application materials considered in holistic admissions. These materials include, but are not limited to, the personal statement, types and duration of extracurricular activities, and indicators of socioeconomic background. These examples represent only a subset of the non-academic components present in the application. However, the results reinforce the need for medical schools to engage in ongoing, collaborative review of admissions requirements and educational outcomes.

## Conclusions

At times of curricular change, it is essential to evaluate whether current admissions requirements align with the demands of the new curriculum. This study examined the relationship between course prerequisites and student performance within an integrated medical curriculum. We demonstrated that biology, chemistry, and inorganic chemistry GPA from four-year institutions correlated with academic performance, whereas the biology, chemistry, and inorganic chemistry GPA from two-year colleges did not correlate with academic performance. Additionally, students who matriculated within a year of undergraduate graduation performed slightly better in the curriculum than those who graduated with at least one gap year. Medical schools should regularly evaluate their admissions requirements in the context of their own curricula to ensure alignment between applicant selection criteria and student’s academic performance.

### Conflict of Interest

The authors declare that they have no conflicts of interest.
